# Combining differentiating and integrating over time to sustain multiple institutional logics: a case study of a higher education institution

**DOI:** 10.3389/fpsyg.2023.1218569

**Published:** 2023-08-24

**Authors:** Ye Jiang, Xiaojun Zhang

**Affiliations:** Academy of Future Education, Xi'an Jiaotong-Liverpool University, Suzhou, Jiangsu, China

**Keywords:** institutional logics, differentiating and integrating, selective bridging, logic hybridization, qualitative case study, grounded theory

## Abstract

To understand how organizations adopt varying configurations of differentiating (i.e., compartmentalizing logics into different subunits or roles) and integrating (i.e., combining logics to highlight synergies) over time to navigate logic contestations and extract logic complementarities for organizational innovation, we conduct a 15-year in-depth longitudinal case study of a higher education institution to examine how it devised innovative hybrid practices to manage and support college student development. By employing the grounded theory method, we develop a four-stage process model of the varying configurations of differentiating and integrating that expounds on how two contested logics are sustained and exploited over time. We assert that selective bridging*—*the instrumental use of one logic enables some practices of one logic to be selectively integrated with the other, while irreconcilable practices remain differentiated to play their respective roles, is vital in allowing organizations to leverage respective advantages in combining differentiating and integrating over time. Further, we show that combining integrating and differentiating features simultaneously transforms contested logics into compatible and complementary, offering a novel way for logic hybridization. These findings advance the understanding of how organizations can differentiate and integrate contested logics over time through a dynamic and paradoxical view, thus being manageable and manipulated for organizational innovation.

## Introduction

Organizations increasingly operate in complex institutional environments governed by multiple institutional logics offering seemingly incompatible prescriptions and proscriptions for actions (Greenwood et al., 2011; Raynard, 2016). Consequently, organizations must devise various strategies, structures, and practices to sustain distinct logics (Battilana et al., 2017). Among these, one novel type pursues the ends of one logic through the means of another, offering a vital approach to cope with multiple logics, especially for organizational innovation and creativity (Battilana and Lee, 2014; Tracey and Stott, 2017). For example, work integration social enterprises create routes to pursue social missions, such as creating employment opportunities for people living at the bottom of the pyramid through commercial channels (Pache and Santos, 2013b).

Recognizing that the juxtaposition of multiple and often contested logics enables organizations to achieve their goals notwithstanding the challenges and complexities created both at the macro-level in terms of governance arrangements and organizational structures (e.g., Ebrahim et al., 2014; Ramus et al., 2017) and at the micro-level concerning actors’ perceptions and identities (e.g., Ashforth and Reingen, 2014; Wry and York, 2017), extensive research has demonstrated various features that differentiate or integrate multiple logics to alleviate their tensions (e.g., Besharov et al., 2019). Differentiating or compartmentalizing logics into different subunits emphasizes the unique means, goals, values, strategies, and practices associated with each logic (Pache and Santos, 2013a), and differentiating can be accomplished through, for example, pluralistic leaders (Besharov, 2014), or separate subgroups (Ashforth and Reingen, 2014). Integrating highlights interactions and synergies, which motivates cooperation or a combination that reflects both logics simultaneously (Ramus et al., 2017). Integrating can be fulfilled via, for example, spaces of negotiation (Battilana et al., 2015) or co-leadership (Gibeau et al., 2020).

Building on this insight and the recognition that differentiating and integrating might develop new competencies and opportunities more attuned to addressing logic tensions, recent research has shown that organizations can shift or adapt their features in response to changing internal and external impacts or turbulences (e.g., Dalpiaz et al., 2016; Hesse et al., 2019), which illustrates the possibilities and values of both differentiate and integrate logics. For example, organizations may have some members who adhere to just one logic, while others adhere to both simultaneously. Besharov (2014) founds that a natural food retailer included front-line workers who were either “idealists” or “capitalists,” endorsing social or commercial logic, but that most managers were “pluralists” who carried both logics simultaneously could draw on their integrative mental models to prevent conflicts when tensions occurred between idealists and capitalists.

Further, the study by Smith and Besharov (2019) demonstrates the value of differentiating and integrating logics within organizational-level features. Their research on the work integration social enterprise unpacks that the leaders created differentiated structures, roles, and external stakeholder relationships devoted to the social welfare or commercial logic, preventing a single logic from dominating. They also employed an integrated organizational structure that pursued the social welfare logic by helping people eliminate poverty by operating commercial businesses, reinforcing linkages and synergies between the two logics. These combined differentiating and integrating features impeded intractable conflicts and mission drift and facilitated productive negotiation between logics.

The potential for organizations to enable sustainable development and innovation depends on their competencies to maintain contested logics over time (Du and You, 2013; Ocasio et al., 2017). However, extant research has overlooked the fact that features of integrating and differentiating can vary over time in an organization, resulting in more complex adoptions and adaptations of logics across organization dynamically (Besharov and Mitzinneck, 2020; Ramus et al., 2021). Thus, we lack a thorough understanding of the dynamics of the organizational combination of varying configurations of integrating and differentiating over time as it evolves and grows and the impact of such dynamics on the capacity of organizations to develop the necessary competencies for effectively navigating logic contestations and extract potential complementarities (Ramus et al., 2021). Besides, while a few studies have shown that contested logic can be hybridized to produce a novel logic, as such, to extract logic complementarities (Battilana and Dorado, 2010; Raynard, 2016), the logic hybridization process at the organizational level received insufficient attention (York et al., 2016).

We thus address these gaps and respond to the call of Besharov et al. (2019, p. 408) to “studies at the organizational level generally depict organizational features as either differentiating or integrating multiple logics, […] Future research can build on these emerging insights to examine whether and how organizations adapt configurations of differentiating and integrating over time.” We ask the following question: *How does an organization adapt varied forms of differentiating and integrating into the process of attaining hybridization between the contested logics over time?*

We conducted an exploratory case study (Yin, 2018) of a Sino-Foreign Cooperative Higher Education Institution (HEI) concerning how it adopts and adapts various configurations of differentiating and integrating during the process of generating complementarities between two contested logics in college student management from 2006 to 2021. First, we develop a four-stage process model that illuminates the varying configurational features of differentiating and integrating as the organization evolves and grows over time. Along with this, contested logic shifts to be compatible and complementary. Second, we observe that ongoing adaptations and combinations of integrating some practices of both logics while differentiating other irreconcilable practices through selective bridging—a constant search for practices that could uphold both logics by using one logic as an instrument to fulfill the enforcement and goals of the other, transforms inter-logic relations from contested to compatible, thus makes logic hybridization possible. Third, paradoxical cognitive thinking and mindset that realize the existence of contradictory yet potentially mutually reinforcing elements simultaneously between contested logics constitute an antecedent that organizations can benefit from combing integrating and differentiating.

Our contributions to the literature are as follows. First, while existing studies have predominantly emphasized relatively static organizational strategies, we provide empirical evidence that organizations can combine varied configurations of differentiating and integrating strategies dynamically to leverage their respective advantages and generate values within organizational-level features. Second, while numerous institutional studies have elaborated on the problematic nature of logic contestations, we demonstrate that individual actors’ paradoxical views and mindsets are essential for organizations to explore processual dynamics in response to logic multiplicity and to unlock the positive potential of logic contestations, which ultimately enable organizations to handle manifold challenges. Third, the ongoing adaptation and combination of integrating, that is, actively seeking distinctions between different logics, and differentiating, that is, exploring synergies and interactions, offers a novel way to achieve logic hybridization.

## Conceptual background

### Characteristics of differentiating and integrating to maintain contested logics

Institutional logics designate “which means are meaningful” and which “means-ends couplets are thought appropriate” (Friedland, 2002, p. 383); that is, institutional logics prescribe behaviors and actions that are considered legitimate and appropriate means to achieve specific goals. Extant studies have adopted various viewpoints to perceive the impact of the juxtaposition of multiple logics in organizations (Greenwood et al., 2011; Ocasio et al., 2017). For example, scholarly research has shown multiple logics as problematic for organizational development, focusing on contestations between logics and claiming that conflicts could be resolved by keeping actors or practices following contentious logics apart (Ashforth and Reingen, 2014). This allows organizations to focus on the distinct demands associated with each logic. Others show potential benefits to integrating divergent competing logics, and synergies and linkages can be created and exploited (Dalpiaz et al., 2016). This motivates these seemingly incompatible logics to cooperate with or complement each other to produce novel solutions by simultaneously representing both logics.

Although challenging, organizations can combine differentiating and integrating at different levels (Ramus et al., 2017). As such, organizations can address contestations between logics and sustain organizational hybridity, thus pursuing novel solutions to organizational challenges. For example, when subgroup actors endorse divergent contested logics (i.e., the differentiating feature), simultaneously adopting integrated organizational features work to reduce the risks of intractable conflicts and promote communication between these actors, fostering a context that cultivates mutual understanding and seeks creative alternatives to satisfy both logics (DiBenigno, 2018; Besharov et al., 2019). Integrating within differentiating features can be accomplished through, for example, boundary spaces (Perkmann et al., 2019) or shared structures and tangible objects (DiBenigno, 2018).

When organizational actors endorse both logics (i.e., the integrative feature), bringing in differentiated organizational features raises actors’ awareness of each logic’s distinctive value, fostering innovative solutions such that both logics play their roles. Ramus et al. (2017) illuminate that the structured interplay between collaboration and formalization helps integrate and differentiate social welfare and commercial logics effectively. Their study shows that Delta can leverage formalization and collaboration to find the appropriate combination of differentiating and integrating while responding to external pressures.

While these studies have demonstrated that organizations can survive and develop by employing particular strategies with differentiating, integrating, or combing both features, we lack thorough understanding in terms of how organizations adopt and combine varying configurations of integrating and differentiating over time as it evolves (Besharov et al., 2019; Ramus et al., 2021). Such a dynamic perspective can support organizations to manage the tradeoff between contested logics over time rather than enacting a single and stable configuration, which may be different in an institutional environment fraught with uncertainty and ambiguity.

### Logic hybridization as a resolution to generate complementarities between contested logics

Institutional studies have recognized that embracing contested logics can benefit organizations, such as broader legitimacy, enhanced efficiency, innovation, or institutional change (e.g., Perkmann et al., 2022). These studies also suggest that complementarities between competitive logics can be extracted through variegated strategies (e.g., Raynard, 2016; Yan et al., 2021). Organizations favor such complementarities and synergies because it generates positive impacts, such as offering social actors more space for discretion (Thornton et al., 2012), and one logic can complement the weakness or shortage of another to further organizational development (Waldorff et al., 2013).

Institutional scholars claim that multiple logics can be hybridized into a new one over time (Ansari et al., 2013; Ocasio et al., 2017) and argue that organizations used to integrate multiple, often antagonistic logics to achieve hybridization (Battilana and Lee, 2014; Battilana et al., 2017). However, while the concept of a hybridized logic has been alluded to, the process of logic hybridization at the organizational level has yet to receive sufficient examinations. For example, Ansari et al. (2013) describe and theorize the processes that result in the emergence of a field-level transnational hybrid logic of climate change that drew from the market, state, professional, and community logics. York et al. (2016) propose that a new hybridized field-level logic emerged due to the reduction of field centralization and the gradual reduction of the incompatibility between the goals of the logics through specific means. Although these two studies elaborate logic hybridization process, they operate at the field level of analysis. Another individual-level study conducted by Fan and Zietsma (2017) elaborates on a recursive process model: agreeing on values, shared learning, and enacting shared values to construct a new and shared governance logic in which organizational members mobilize social emotions, moral emotions, and emotional energy.

Thus, this study argues that the processes through which organizations attain a novel hybridized logic deserve to be examined further. Specifically, this study intends to explore how organizations adopt varied differentiating and integrating configurations over time to achieve logic hybridization, which enables organizations not only to alleviate logic incompatibility but also to extract logic complementarities, thus to be more innovative and sustainable (Dalpiaz et al., 2016).

## Methods

We adopted an exploratory approach following the tradition of constructivist grounded theory through an in-depth case study (Charmaz, 2014; Yin, 2018).

### Research setting: a Sino-Foreign Cooperative HEI

We chose T-University for the case study. T-University (pseudonym) was co-founded by top-ranking universities in China and the UK in 2006. It is neither a traditional Chinese nor a British university. It endeavors to establish a distinct educational model by combining best practices from its parent institutions without duplicating either.

There are two logics guiding T-University in managing its students. It endeavors to explore its approach to college student management by constantly advocating traditions rooted in local Chinese society and practices borrowed from the West. Drawing from its UK counterpart, T-University conceived its students as mature adults with responsibility, consciousness, and a sense of initiative, so that they could manage and arrange their academic studies and personal lives independently.

However, the students exhibited poor self-discipline, encountered severe maladjustment, and faced psychological problems since they were accustomed to a spoon-feeding learning style and passively received indoctrinated knowledge from their teachers before entering university (Wu, 2014). T-University has also deployed traditional Chinese universities’ student management practices, such as setting rigid rules and regulations (Zhu, 2016). UK traditions and Chinese practices are embedded in the rules, norms, and practices that constitute T-University’s organizing principles and can provide cognitive and practical concepts and templates for organizations and individuals to perform tasks and organize behaviors (Thornton et al., 2012). Thus, the institutional logics approach is consistent with our case, and from the institutional logics perspective, the UK traditions and Chinese practices embody two different institutional logics. Thus, T-University offers a meaningful and well-suited research context for addressing the research questions and provides multiple opportunities to observe the phenomenon of interest.

### Data collection

This study gathered data for 2006–2021 and relied on archival documents and semi-structured interviews with open-ended questions. Media news and articles were used to complement and triangulate data. The detailed sources and uses of the data are summarized in Table 1.

**Table 1:** Details on data collection.

Data sources	Use in the analysis
Semi-structured interviews (40 interviews, 37 participants, and 428 pages of data)	
Each interview lasted between 45 and 120 min.	(a) Insights on the philosophy, motivations, and tactics T-University adopted for college student management. (b) The critical developmental processes in college student management at T-University, especially the key events and activities and challenges and problems T-University faced. (c) Coded for the differentiating and integrating strategies that T-University adopted over time and their subsequent impacts, as well as observing the relationships between the two logics during different developmental stages.
Archival documents (768 pages of data)	
*(1) University Policies and Regulations (111 pages)* For example, Academic Policies and Procedures Handbook; Regulations on Student Administrations; Mitigating Circumstance Policy, Policies on Student Attendance and Engagement; Policy on Student Attendance, Resit, and Program Participation (selected among specific modules); Policy on Student Conduct and Discipline, and so on.	(a) Identify the policies and regulations drafted for college student management at T-University. (b) Observe specific means and tactics T-University adopted to regulate student behaviors. (c) Coded for the differentiating and integrating strategies that T-University adopted over time and their subsequent impacts, as well as observing the relationships between the two logics during different developmental stages. (d) Identify critical events and activities at T-University, which may generate significant impacts.
*(2) Handbooks (6 handbooks, 468 pages)* For example, Student Development Advisor Handbook; Buddy Program Handbook; External Mentor Handbook; Selected Module Handbook; Bounce Back Program Handbook; Student Club Support Center Handbook.	
*(3) Other Useful Documents (189 pages)* For example: Confidential emergency reports, Reports of the Exploration and Practices for the Student-Centered Education Model, etc.	
Media news and articles (55 pages of data)	
*Website news and articles (15 news items)*	(a) Critical events that happened between 2006 and 2021. (b) Articles concerning those practices and strategies adopted by T-University for student affairs.
*WeChat articles and information (10 articles)*	

#### Semi-structured interviews

We conducted 40 intensive semi-structured interviews with 37 participants, and three of the participants were interviewed in two rounds (one Senior Development Advisor and two Deputy Heads) from September 2021 to December 2022. The three participants have worked at T-University for over 10 years, and we seek to seek more information on why any subtle changes happened and how, as well as confirm with them our understanding of the college student management development process at T-University. During the interview, participants were invited to describe retrospectively, from their perspective, the key events and decisions that characterized T-University’s formative years. We aim to elicit how staff at the Center for Student Affairs understood and were involved with the different logics in their daily work and the practices used to manage students during distinct developmental processes.

The interviewees included professional supporting staff from the Center for Student Affairs, who was responsible for student development and non-academic issues; academic staff who acted as academic advisors to help resolve academic issues and focus on the academic performance of students; and staff in management positions who had a profound understanding and knowledge of the development of student management practices at T-University. The senior management team members, such as vice presidents and the executive president, were also included to gather information on the rationale and mission of the student management and development practices.

To minimize respondents’ biases (Patton, 2002), we designed a semi-structured interview protocol that we adapted to the characteristics of different informants and refined over time as the research progressed and theoretical constructs emerged. We identified participants through snowball sampling during interviews, primarily concerning their involvement in these processes.

We continued interviews until we reached an in-depth understanding of the phenomenon under exploration. New interviews provided no fresh and relevant information for developing a new theory; we had reached theoretical saturation (Charmaz, 2014). All interviews were conducted face-to-face and lasted between 45 and 120 min. The recordings were tape-recorded and transcribed verbatim, generating 428 data pages for analysis.

#### Archival documents

We also relied on archival data. We used official university policies and regulations, as well as internal handbooks and reports pertaining to students, such as the Academic Policies and Procedures Handbook, Regulations on Student Administrations, and so on (see Table 1 for details). These archival documents comprise 768 single-spaced pages of data.

#### Media news and articles

We looked up milestones in the development of student affairs at T-University and regularly followed and searched the media for news and articles, mainly on news websites and WeChat. Together with the interviews and archival documents, we also used these events, news, and articles to identify critical events that substantially transformed the previous practices, structures, and activities of T-University. These critical events are “critical junctures” that “durably transform previous structures and practices” (Sewell, 1996, p. 843). We constructed a timeline of events concerning feedback from informants.

### Data analysis approach

To conduct the analysis, we moved iteratively between data, relevant literature, and the emerging theory (Gioia et al., 2013). Through constructivist grounded theory (Charmaz, 2014), we moved from raw data to categories and themes. The analysis proceeded through three steps.

#### Stage 1: identification of multiple institutional logics in T-University

**Figure 1:**
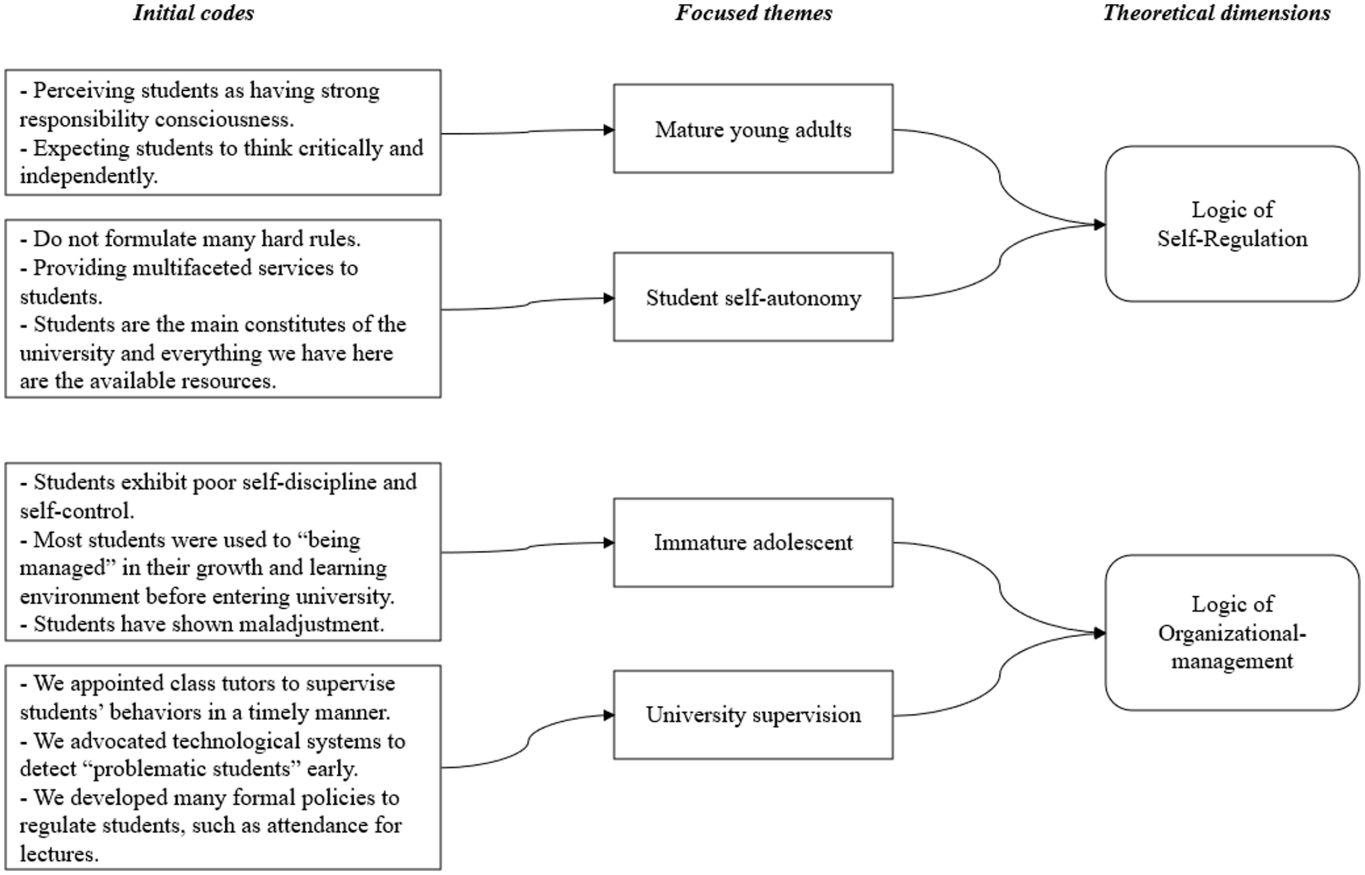
Structured coding of institutional logics.

We first identified the logics that unfolded in college student management at T-University by following the inductive analytical method (Reay and Jones, 2016), which adopts a grounded approach and starts with the raw collected data. We read and coded the interview and archival documents and clustered the themes that reflected the behaviors, norms, and beliefs observed in the data, which were consistent with those of logic. The coding structure is illustrated in Figure 1.

#### Stage 2: identification of critical events and coding of the developmental stages

In the second stage, we began our analysis by identifying the key events and activities at T-University from 2006 to 2021 (see Table 2). We established a timeline of critical events to analyze process data (Langley, 2007), such as appointing a new presidential team, establishing new position changes in organizational structures, and implementing new programs. We confirmed these events using key informants to ensure validity and accuracy. We obtained four partially overlapping periods (2006–2009, 2009–2013, 2013–2016, and 2017–2021), which we used as the units of analysis (Eisenhardt, 1989).

**Table 2:** Timeline of critical events that impacted college student management at T-University (2006–2021).

Period	Events	Year
Period 1 (2006–2009)	The Inaugural Executive Vice President of T-University had been appointed from a UK university (the British parent institution of T-University).	2006
	The leader in charge of student affairs at the functional level was recruited from a traditional Chinese university.	2006
	The new university leadership team.	2008
Period 2 (2009–2013)	A centralized university-level student service center had been established.	2009
	Establish and implement student-centered and interest-driven educational modes.	2009
	Official policies, regulations, and procedural principles drafted to standardize college student management.	2010
	Class and class tutors were established for year 1 students.	2013
Period 3 (2013–2016)	Welfare advisors were appointed.	2014
	New programs initiated to support college students’ management.	2014
	Application of informational and technological systems.	2015
	New policies and rules drafted and initiated to regulate and supervise students’ behaviors (e.g., the attendance policy)	2016
	Development Advisors (DA) were appointed on a part-time basis.	2016
Period 4 (2017–2021)	Full-time Development Advisors (DA) were appointed.	2017
	10 Virtual functional teams were established within the DA team.	2018
	New Vice President was appointed, responsible for the Centre for Student Affairs.	2019
	Set up the Student Party Affairs team.	2020
	WINGPLUS platform established.	2020
	Close collaborations were initiated with the academic department.	2021
	Student party branch set up in each academic department or academy.	2021

#### Stage 3: identifying integrating and differentiating strategies for enacting the logics in different periods: practice-level analysis

Through our interviews, we identified three practices within T-University (Smets et al., 2015; Cappellaro et al., 2020) that were core for college students’ management: “structural” practices, which primarily referred to the setting up of essential roles, teams as well as any other structural changes; “programs and policies” practices, which centered on the programs and policies specifically designed for college students management; and “mental support” practices, which concerned students psychological health and mindset development. These practices were especially central to the unfolding process regarding how the two logics were enacted at T-University and their changing relations during this process.

**Figure 2:**
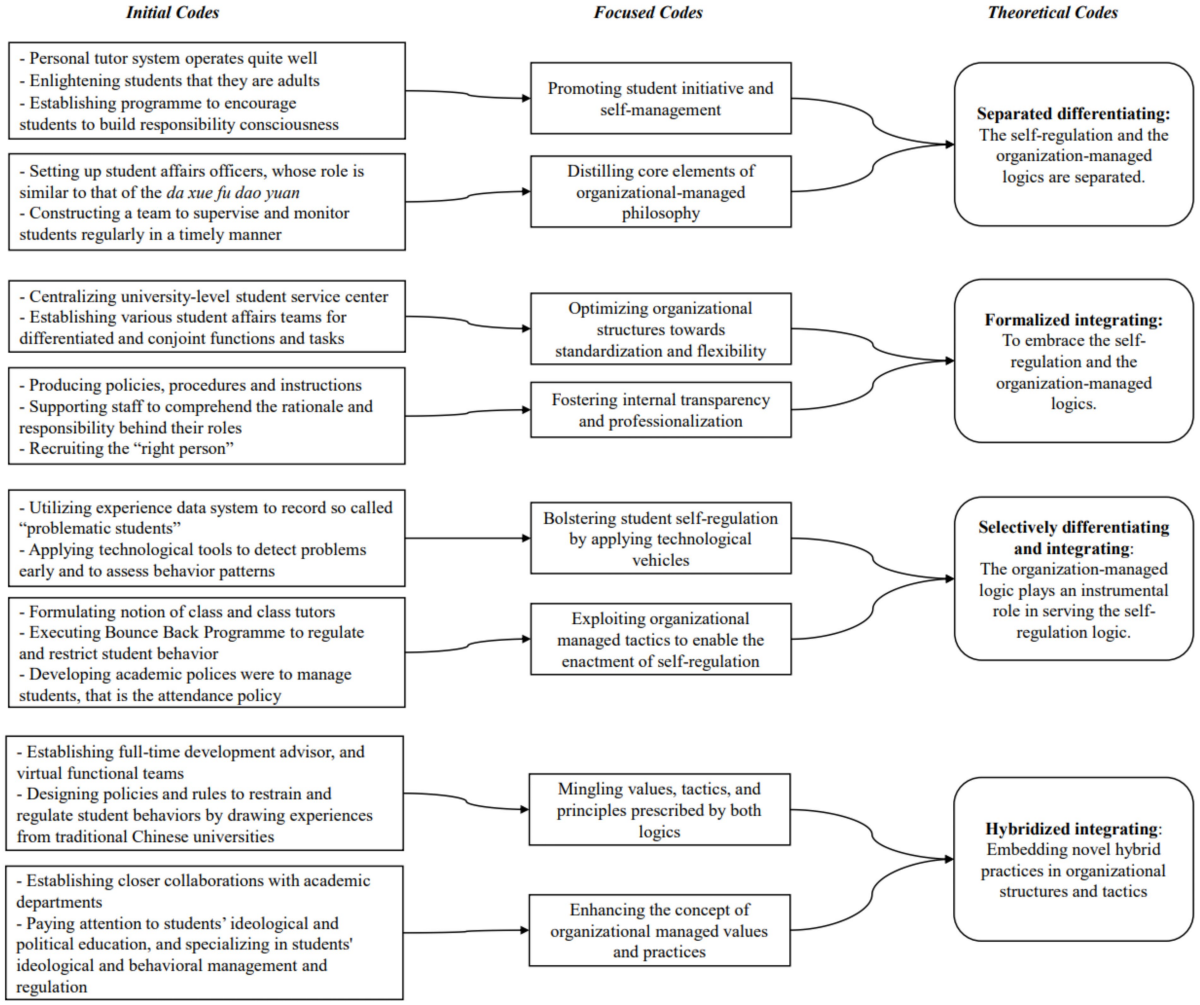
Structured coding of data.

The collected data were analyzed to understand how the three core practices were enacted at T-University. We first identified first-order codes using the participants’ frequently appearing initial words and languages to categorize and synthesize the data (Charmaz, 2014). We further abstracted these clustered practices that could be allocated to integrating, differentiating, or both into aggregated theoretical dimensions. Based on the results inductively obtained, this study theorized that these four phases unfolded a processual relationship wherein a dynamic configuration combined differentiating and integrating features. Figure 2 summarizes the data constructs across the four stages.

To ensure the trustworthiness of the analysis (Lincoln and Guba, 1985), we triangulated across interviews and massive archival data. This served to reduce potential retrospective bias in the analysis. We wrote thick memos during and after data collection and analysis to integrate various data sources and capture the rich context over time (Langley, 2007). We also conducted member checks to secure the fidelity of our interpretation of different events.

## Findings

### Multiple logics identified at T-University

Following what was inductively generated from our data (Reay and Jones, 2016), we identified two logics that guided college student management at T-University. The *student-led logic* claimed that college students should be perceived and enlightened as young mature adults with independent personalities and strong responsibility consciousness and that students themselves can fulfill self-management and self-discipline and think critically and independently. This logic prescribes students to be self-regulated, self-managed, and self-governed, emphasizing student autonomy and mobilizing their initiatives. T-University did not make rigid rules to regulate student behavior. Instead, it provides multi-faceted services and guidance and creates international platforms for its students.

However, most students were part of a growth and learning environment where they were “managed and instilled” before they enrolled in T-University. Thus, they embodied maladjustment and misunderstanding in the educational philosophy, organizing principles, and teaching modes at T-University. Thus, T-University also employed various roles, tactics, programs, and formal policies to supervise students, which shows the *organization-led logic*. This logic treats students as immature adolescents with poor self-control and self-discipline ability and believes that the university should be responsible for managing students. The means prescribed by organization-led logic are that students should be supervised by their teachers, institutions, and higher governmental authority, such as rigid rules, which highlight discipline and obedience.

### Varying configurations of differentiating and integrating in sustaining multiple logics

We now present the four periods in which varying configurations of differentiating and integrating strategies have been adopted, and the relationships between the logics change over time. Finally, a hybridized logic emerges.

#### Period 1 separated differentiating (2006–2009): logics are compartmentalized—“we are doing things in different units!”

As T-University is a Sino-Foreign Cooperative University, its college student management must consider the practices in China and the UK. Thus, it inevitably faces plural demands of multiple logics – student-led and organization-led logics, which are rooted in traditional Chinese universities and Western universities, respectively. We now elaborate on how the logics are differentiated in practices during its first developmental period.

Since its establishment in 2006, university leaders have created differentiated formal structures and roles, programs and policies, and mental support dedicated to student-led or organization-led logic. Regarding structural arrangements, T-University advocated the British personal tutor (PT) system as an essential system to support students. According to this system, a personal tutor is responsible for five to eight students for their academic and non-academic issues, and it is the students’ responsibility and freedom to look for assistance from their PT, intending to “*mobilize students’ initiative and encourage students to think independently, thus fulfill self-management*” (Vice-President #1).

For programs and policies, T-University initiated several programs, such as the Buddy Program, executed as splendid senior students communicating and guiding first-year students to encourage them to realize self-management and build responsibility consciousness continuously. Regarding mental practices, after students enrolled in T-University, “*they were enlightened as young adults with mature minds to engage in self-management and self-discipline*” (Director). Rather than monitoring and management, the roles of university and personal tutors centered on providing professional services and guidance, resources, and facilities to assist students’ academic and overarching development.

These practices embodied student-led logic aim to promote student initiative and self-management, as the vice president stated:

“*In most cases, we do not make rigid or hard rules for our students. We encourage them to mobilize their initiatives to seek assistance from their tutors. They are also responsible for achieving study-life balance, handling other self-management issues,* etc.” (Vice President #1).

Conversely, organization-led logic also held its position. From a structural perspective, T-University also established the Student Affairs Office and the role of a university counselor (similar to *da xue fu dao yuan*) was proposed at the functional level. The Director of Student Affairs stated: “*This office was completely separated from the personal tutor system.*” Although the responsibility of the university counselor was ambiguous at this point, officers in this position supervised and monitored the students’ behaviors. Similar to the practices of programs and policies, T-University sets regulations on student administration, which aims to cultivate students’ balanced development in moral, intellectual, physical, and aesthetic aspects, and their behaviors to be consistent with these regulations. Regarding mental support practice, T-University regularly organized diverse mental support seminars “*aimed to build students’ sense of belonging and guide them adapt to university life, and students have to attend no less than twice per semester*” (Manager #2).

The Director of Student Affairs at T-University explained these differentiated but co-existing practices.

“*In the beginning, the personal tutor worked effectively to encourage self-autonomy, and most students had a solid connection with their PTs. However, as traditional Chinese students, they are used to “being managed.” So, we established the position of a university counselor in the Student Affairs Office, similar to da xue fu dao yuan in traditional Chinese universities, to pay close attention to students. I believe we did the right thing!”* (#Director).

Overall, we observed that student-led and organization-led practices co-existed but were differentiated (Goodrick and Reay, 2011) such that each plays its respective role in guiding student management. This differentiating strategy enabled T-University to maintain distinctive logics by embodying logics in separate routines, practices, structures, and carriers of each logic are likely to value each logic’s distinct value. However, many students started to experience difficulties in their mental and environmental adaptation, which manifested in several ways, such as psychological problems, repetitively failing exams, or even dropping out. Thus, the differentiating strategy only worked for some time, and continuous student problems make university leaders have to develop further possible solutions.

#### Period 2 formalized integrating (2009–2013): conflicts are triggered—“how can you do that? why do you do that?”

In late 2008, the senior management team underwent tremendous changes, and a new presidential team was appointed, significantly altering the university structures. The new presidential team established the Centralized Student Service Center (CSSC) in 2009, which aimed to improve the overall efficiency and student service quality. This change has enabled the comprehensive inclusion of student- and organization-led logics in the CSSC. Following this, as one team leader described, “*well-defined formal rules, responsibilities, and procedures were proposed to perform specific tasks and activities*.” However, this formalized strategy attempted to integrate both logics into practices, but it failed and triggered conflicts between actors who endorsed distinct logics.

In terms of the structural practices prescribed by the student-led logic, official professional student affairs supporting teams, such as mental health and development, student organization support, and art education, were constructed for differentiated functions and completing conjoint tasks, which all served as supporting units that were available for students to seek assistance. “*These teams were set up to provide multi-facet services, platforms, and resources for students to use, but it is still students’ responsibility and initiatives to look for support*” (Manager #2).

At the centralized level, massive policies, formal procedures, and instructions were drafted and produced to formalize and standardize student management affairs. As one Deputy Head stated: “*T-University is a brand new and unique university, and we must learn and explore things ourselves. We cannot imitate anyone, so we have to develop our standardized process and appropriate strategies to support our student management and development.”* (Deputy Head #1).

Thus, in this phase, student-led and organization-led logics were formalized into a centralized unit by optimizing the organizational structure and fostering internal transparency and professionalization (Ramus et al., 2017). Both logics’ values, beliefs, and practices are codified and reflected in regulations, policies, and positions that clearly define and expound student responsibilities, procedures, rewards, and punishments.

Straight after this, contradictions were occasioned. While applying and implementing both logics in the centralized unit, “*conflicts from distinct stakeholders appeared and persisted during this period*” (Team Leader #4). Regarding the practices of programs and policies, the student-led logic prescribes that enough space and greater discretion should be given to students for their academic and non-academic affairs. The student affairs officers who advocated this logic in their daily practice dismissed the organization-led principles as “*rigid, largely centered on risk avoidance, and bureaucratic, regardless of the students’ intrinsic needs*” (Development Advisor #2). Therefore, it was treated as unprofessional and unfit for T-University’s student-centered and interest-driven concepts.

Contrarily, regarding structural practices, some student affairs officers’ responsibilities became more articulate under organization-led logic. For example, each was allocated a fixed number of students, and the officer had to talk to them regularly to supervise their progress. Concerning programs and policies, “*Lots of policies, procedures, and instructions have been produced and drafted to standardize the management of students affairs, and to complement the informal procedures*” (Manager #3), which aimed to restrain students’ behaviors better. Under such circumstances, some student affairs officers, especially those from traditional Chinese universities, were inculcated with organizational-management logic. They claimed that “*student self-autonomy is not always possible and feasible because our students are Chinese*” (Development Advisor #1).

Thus, both logics were carried by different actors, who actively tried to defend and justify their practices and tactics and challenged others as ineffective. The contradictions also showed that both logics championed by different organizational groups led to the emergence of internal tensions (Pache and Santos, 2010). Further, student problems continue to occur as their prior inertia to organization-led practices manifests severe difficulties in adapting to student-led practices. Corresponding with the dissatisfaction from students’ parents, university leaders and student affairs officers were alerted to the unsustainability and infeasibility of the contested relations between student-led and organization-led logics. Thus, T-University began to explore new student management methods for its sustainable and practical development in the long term.

#### Period 3 selectively integrating and differentiating (2013–2016): logics are selectively bridged, and compatibility is constructed—“we are trying to exploit and adopt this”

To resolve the contradictions, T-University focused on practices that may differentiate and integrate to prevent intractable tensions and create linkages and synergies between student-led and organization-led logics. As such, the organization-led practices are constructed as compatible (rather than opposed) with the student-led alternative. As such, having actors integrate some selected practices from both logics dexterously alleviated tensions while differentiating remaining irreconcilable practices enabled some elements of both logics to play their respective roles in managing college students.

First, student affairs officers needed to understand and realize the strategic usefulness of both logics and collaboration to exploit benefits. University leaders, acting as conveyors to disseminate the values and advantages of the tactics stipulated by both logics, have a significant impact. Contrarily, student affairs officers also began to reconsider the student management challenges they encountered and worked toward reflection and exploration. This change in understanding was significant, as it was a prerequisite for the next step. After that, T-University devoted itself to exploiting organization-led practices to enable and facilitate the enactment of student self-autonomy and self-management. A team leader stated this transformation: “*We began to think about and reflect on why our students had so many problems and maladjustment after enrollment. We realized that we probably needed to use the ways and strategies they were used to when they were in high school, thus to support them fulfill self-management principles gradually.”* (Team Leader #1).

Concerning structural practices, in 2013, T-University established the concept of class and the role of a class tutor. One deputy head stated: “*Consistent with the values of organizational-management logic, class tutors arranged class meetings and one-to-one student-tutor meetings regularly to detect students’ problems promptly*.” In addition, T-University established a technological data management team, and the technological systems they applied were a significant tool to monitor student behavioral patterns. Using a technological information system, “*students’ university resource utilization rate, student activity rate, or their spatial data on campus were observed*” (# Team Leader 2). The aim was to detect student problems early, such as meager attendance rates.

In terms of programs and policy practices, several vital programs were initiated, among which the most typical one was the Bounce Back Program (BBP). It was a structured and experimental support program that integrated techniques of psychology and pedagogy and sought to assist students in enhancing their self-efficacy, self-discipline, and self-management abilities. “*The habit formation module primarily comprised early morning assembly, class punch-in, daily exercises, self-study, and so on*” (Team Leader #1). All daily tasks were subject to strict and timely supervision by student affairs officers.

Academic policies have also been refined and revised to regulate student behaviors, such as revisions in the module specification and attendance policy, which state: “*It was the students’ responsibility and willingness to attend lectures and tutorials in the past. However, the new academic policy clearly states that students must attend all scheduled lectures and tutorials for their programs*” (Academic Advisor #4).

Finally, regarding mental support practices, the mental health support team offered individual and group psychological counseling services to students who could not adapt to university life and had psychological issues, thus repeatedly failing exams. Those “*problem students must attend this activity or talk to the counseling officer regularly*” (Senior Development Advisor #2).

The practices mentioned earlier regarding structural programs and policies and mental support prescribed by organization-led logic acted as the means to support the implementation of student-led alternatives at T-University. This is because the University aims to cultivate students’ autonomous competence and innovative and critical skills through students’ self-management and self-discipline.

Student affairs officers who advocated respective logics changed their working means and mechanisms to accommodate each other. This study observed that the selected practices from the organization-led logic were adopted as an instrumental tool to bolster the implementation and development of student-led alternatives at T-University. Rather than obeying pre-specified templates or procedures in Period 2, they did so selectively and flexibly by following their judgments rather than following a pre-stipulated template while facing different cases. This strategy is defined as *selective bridging*; some organization-led practices were selectively chosen and bridged to enforce student-led alternatives, which was essential to accommodating the contradictory logics.

In this process, some selected organization-led practices were leveraged as compatible with and supportive of student-led alternatives to reap the benefits of pertinent aspects of one logic into the enactment of another. The synergies and interdependencies between the two competing logics were recognized and realized in this regard. Consequently, the carriers of both logics deliberately changed their working ways to think and work together to find out the “*best solution to resolve students’ problems and how it can be effectively achieved*” (#Director).

Selective bridging creates an environment that facilitates mutual understanding and generates opportunities for confronting and working through tensions between logics. As a result, the relationship between student-led and organization-led logics shifted from contested to compatible. In this way, integrating some practices and differentiating others simultaneously not only prevented unremitting conflicts but also fostered synergies, and consequently, newly integrated hybrid practices started to emerge, triggering the next phase.

#### Period 4 hybridized integrating (2017–2021): logics becoming complementary—“we make it, and we create our unique model!”

At this final stage, the student management mode enhanced and emphasized the concept of organization-led values and practices. It proceeded toward comprehensively mixing and integrating the values, tactics, and principles prescribed by both logics. Eventually, the two logics became complementary and mutually reinforcing, and a new hybridized logic emerged and was actively produced and infused into organizational practices and arrangements.

A novel hybridized logic arose through the overarching hybridization of the practices and principles stipulated by both logics. Regarding structural practices, one important initiative was the establishment of full-time development advisors (DA) and 10 virtual functional teams. The development advisor worked in a hybrid role that integrated the essence of both logics. The president stated: “*The role of development advisor is a new exploration of college student management under the Sino-Foreign Cooperative education mode. It is a working concept and method that has absorbed the essence of domestic traditional universities and Western universities*.” The DAs acted as an essential manifestation of this hybridized logic that not only gave students spaces and discretion (i.e., prescribed by student-led logic), for example, “*by encouraging and mobilizing students’ initiatives to be responsible for their college lives and studies*” (Development Advisor #3) but also supervising with an appropriate extent to avoid potential risks (i.e., prescribed by organization-led logic), for example, DAs began to “*collaborate closely and proactively with academic departments to gain a comprehensive understanding of students, especially for their academic performance*” (Senior Development Advisor #1).

Moreover, the 10 virtual functional teams were established to, for example, complete tasks such as “*students’ services affordance, students’ platforms maintenance, and improvement, aligning with external useful stakeholders to support students’ development*” (Manager #1), such as external industry tutors, and students’ emergencies settlement and so on. These 10 teams worked to implement this new hybridized logic that regulates and gives students enough space for growth.

In terms of programs and policies practices, by drawing from the practices of traditional Chinese universities, T-University’s policies and rules were designed and drafted to formally “*restrain and regulate student behaviors when they were not able to manage themselves*” (Deputy-Head #3). For mental support practices, student management also began to emphasize ideological and political education and specialize in student ideological and behavioral management and regulation, which aims to impact students ideologically. Hence, practices were adopted to enhance the importance of “*managing students rather than laisses-faire*” (Development Advisor#5).

Thus, the empirical data revealed that while the student-led and organization-led logics were integrated to generate a new hybridized logic, thus they shifted to complement each other.

This study noticed that the final integration and embeddedness process was accomplished through purposive actions and managerial intervention; that is, the hybridized novel logic and practices involved more deliberate and managerial actions to infuse the foundations and values of the new hybrid logic into university practices, organizational structures and procedures, and governance arrangements. The relationship between both logics in this process shifted from being compatible to complementary, a prerequisite for the hybridization process. This final stage also enabled T-University to secure endorsement and satisfaction from stakeholders, such as students and parents, and achieve effective student management.

**Figure 3:**
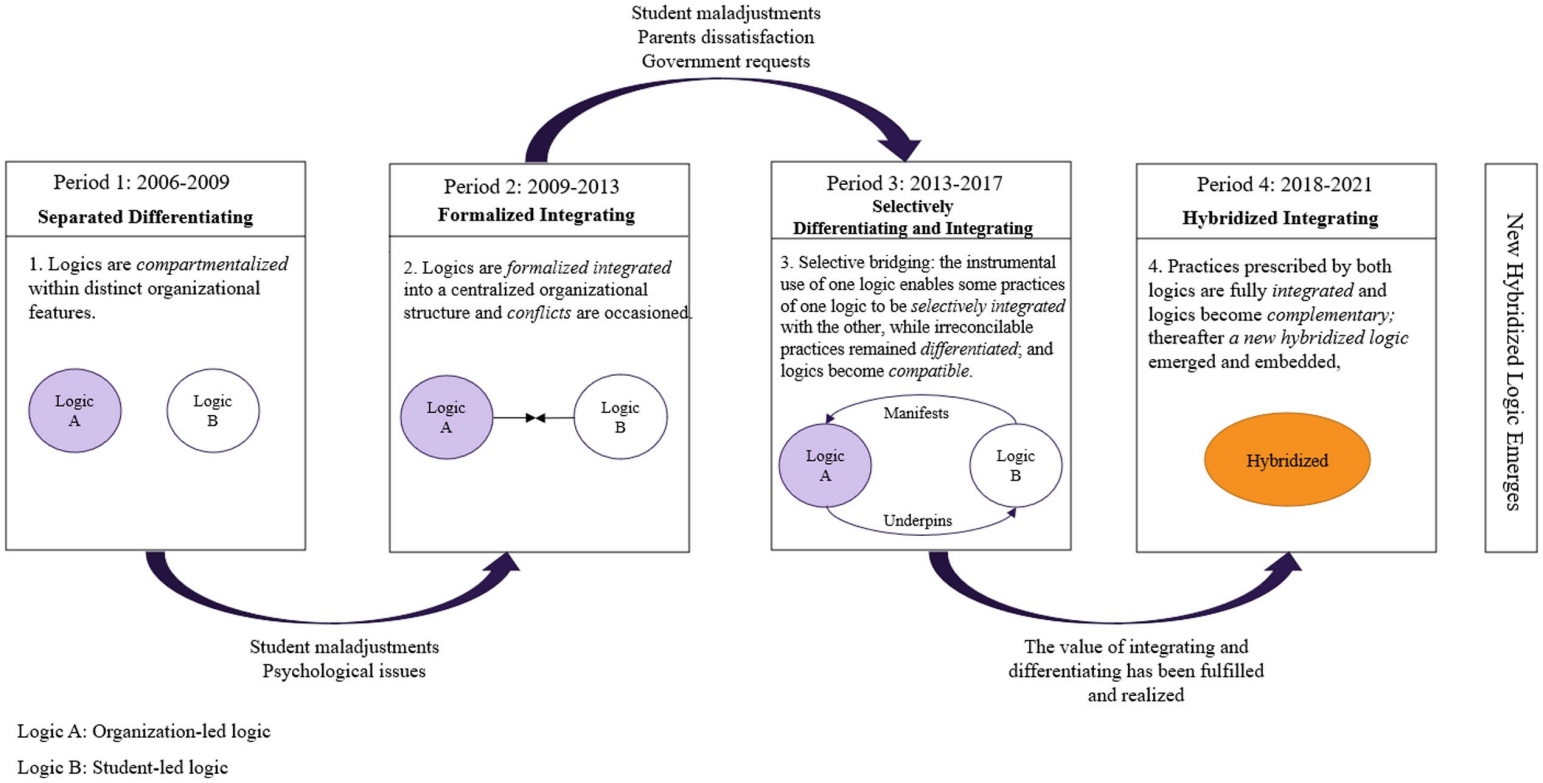
Varying configurations of differentiating and integrating in sustaining multiple logics over time.

Overall, this developed an overarching four-stage process of varying configurations of differentiating and integrating that led to a new hybridized logic (see Figure 3). We conceptualized these four-stage processes in chronological order, with each process constituting an indispensable part.

## Discussion

### Theoretical implications

We demonstrate that adopting and adapting varying configurations of differentiating and integrating allows the organization to provoke an ongoing and processional response to engage with the contested yet potentially mutually interdependent logics. During these dynamic processes, the inter-logic relations transform from competitive to complementary. These findings contribute to the extant literature by elaborating that the dynamic and paradoxical perspectives to understand organizational hybridity is significant, that is, how organizational hybridity emerges and evolves as well as how it is embedded in particular institutional contexts (Besharov and Mitzinneck, 2020).

Extant studies on handling multiple institutional logics emphasize more static responses (Smith and Tracey, 2016). For example, scholars argue that multinational companies face a “particular form of institutional complexity” or “institutional duality” (Faulconbridge and Muzio, 2016, p. 91) when operating its subsidiary in a new country. Scholars depict these pressures as relatively and predominantly stable and claim that MNCs could adopt specific “business models, practices, and structures established as standard” (Kostova et al., 2008, p. 998–999). Similarly, institutional scholars also demonstrate that organizations adopt various strategies that tend to neglect the fluidity and dynamics in managing multiple logics (Holm et al., 2017). For example, Battilana and Lee (2014) explicitly encourage institutional scholars to move from depicting established and fixed hybrid organizations that live with competing logics and examine hybrid organizing as variable and adaptable processes.

Organizations have adopted a variety of strategies to resolve tensions or harvest potential mutually reinforcing benefits from logics; however, most extant studies illustrate that organizations and their members employ strategies that differentiate or integrate these logics, or combine both features (Battilana et al., 2017; Besharov et al., 2019). Consequently, we need a more thorough understanding of how organizations can adopt varying configurations that differentiate and integrate contested logics over time to navigate tensions effectively (Besharov and Mitzinneck, 2020).

We address these gaps and add to the extant literature by adopting a *dynamic* view in maintaining the two contested logics over time and developing varied configurations of differentiating and integrating as the organization grows and evolves to show how tensions are experienced as well as the competencies of organizations to enact and manipulate different responses to deal with logic tensions over time. Further, we illuminate that a *paradoxical* view and mindset are essential for organizations to explore processual dynamics in response to multiple logics and to unlock the positive potential of tensions (Zhang et al., 2015). This paradoxical view perceives competitive logics as a double-edged sword, potentially eliciting innovation and peak performance but also stimulating anxiety that may raise tensions and counterproductive results (Miron-Spektor et al., 2018). This study thus indicates that in the face of the negative consequences generated by contested logics, a paradoxical view and mindset are essential in fueling organizational sustainability and innovation.

Furthermore, based on the varying configurations of differentiating and integrating over time, this study offers a novel way to achieve logic hybridization. This logic hybridization process is significant because it guides the setup and adjustment of organizational practices, strategies, and governance arrangements (Ansari et al., 2013). However, the organizational-level logic hybridization process is still being determined in the literature (Thornton et al., 2012; York et al., 2016). This study observes a logic hybridization process that contributes to this strand of research by differing the logic hybridization process from previous studies.

In this study, the conflicts between the carriers of two logics were occasioned since integrating in the formalized stage (2009–2013). While formalization used to be perceived as an effective way to enable different logics to collaborate fruitfully by providing clear guidelines and signposts (Battilana et al., 2015) and accelerating decision-making (Ramus et al., 2017), our study illuminates that formalized integration can also become a restraint that hinders productive collaboration between logics. Indeed, during this stage, the conflicts between the representatives of the two logics were triggered because formalized integration impeded organizational actors’ flexibility and adaptability within their working tasks and reduced the organization’s competence to change and deal efficiently with unexpected circumstances (Davis et al., 2009).

We find that it is through means that comprising both integrating and differentiating simultaneously offers a novel way to transform contested logics into compatible and ultimately makes logic hybridization possible. Thus, the formation of hybridized logic depends upon the compatibility and complementarity between contested logics. This process was accomplished through purposeful improvisation by organizational actors, in terms of which aspects of the logics they deployed and the purposes for which they employed them to complement the implementation of another logic.

### Managerial implications

It is believed that the experience of conflict could foster an explorative and insight-oriented mindset to search for novel solutions (Leung et al., 2018). Alerted by these contradictions and problems, university leaders realized it was insufficient to formalize the two logics together to integrate them. They redeveloped a particular strategy, that is, selective bridging, exploiting some practices of one logic to fulfill the enforcement of the other. In other words, they integrated some practices of the logics while differentiating others to settle, and as such, the competitive logics became compatible. These integrated practices through the instrumental use of one logic generate shared responsibility for and routine engagement with the multiple elements motivate organizational members to find ways to work together.

This study implied that organizational leaders’ “paradoxical cognitive” thinking—a mental template that encourages people to recognize and embrace the simultaneous existence of contradictory elements is vital for organizational innovation (Smith and Tushman, 2005; Leung et al., 2018). The instrumental use of one logic as the means enables paradoxical cognitive thinking that allows apparent contradictory logics to collaborate toward mutually reinforcing and compatibility (Miron-Spektor et al., 2018), which encourages organizational leaders to grapple with rather than avoid strategic contradictions and to select particular elements of one logic further to develop novel solutions. Thus, by framing how organizational leaders approach contradictions, our study is significant and critical in understanding how organizational members can develop such paradoxical cognitive frames and enhance creativity and innovation (Besharov et al., 2019).

Moreover, formalization, that is written formal policies, rules, and instructions that specifically state the responsibilities and procedures for the fulfillment and accomplishment of takes and activities (Canales, 2014), used to be perceived as an effective way to enable different logics to collaborate fruitfully by providing clear guidelines and signposts and accelerating decision-making (Ramus et al., 2017), our study implies that formalized integration can also become a restraint that hinders productive collaboration between different parties. Indeed, in our study, the conflicts between the representatives of the two logics were triggered because formalized integration impeded organizational actors’ flexibility and adaptability within their working tasks and reduced the organization’s competence to change and deal efficiently with unexpected circumstances (Davis et al., 2009).

## Conclusion

This study addressed how the organization adapts configurations of differentiating and integrating in attaining hybridization between contested logics over time. By studying the evolution of college student management in a Sino-Foreign Cooperative higher education institution, this study demonstrates how the university adapts and combines varied means of integrating and differentiating to develop innovative practices to support college students’ development. These findings show that organizations are not passive recipients but think paradoxically to devise out-of-the-box solutions to problems and challenges. They act purposefully and strategically to achieve their goals and defend the interests of the relevant stakeholders. Multiple institutional logics, irrespective of compatibility, provide sources for organizations to comply selectively. While facing multiple institutional logics, this study provides empirical shreds of evidence that the ways of organizations implement the logics are more nuanced and complex than simply adopting or resisting, as well as an ongoing process of the emergence of novel hybrid practices by extracting and harvesting complementarities between contested logics dynamically and paradoxically.

This study has limitations but also unlocks pathways for future research. First, being adopted a dynamic perspective where we intended to capture the phenomena of interest over time, we had to rely on the memories and stories of our informants, i.e., the inherent retrospective bias of interviews regarding past events and issues. However, we had the opportunity to interview many faculty who had worked there for over 10 years or had worked there since its establishment. Second, while we conducted a single, in-depth case study, it may have the issue of generalizability. However, this limitation also offers avenues for future research. For example, further research may want comparative studies in different settings across countries. Third, we focus on the questions of “how”; thus, we may suggest future research could investigate what factors may impact organizations’ and individual actors’ choices in dynamically enacting different strategies.

## Data availability statement

The original contributions presented in the study are included in the article/supplementary material, further inquiries can be directed to the corresponding author.

## Ethics statement

The studies involving humans were approved by the XJTLU Research Ethics Committee. The studies were conducted in accordance with the local legislation and institutional requirements. The participants provided their written informed consent to participate in this study. Written informed consent was obtained from the individual(s) for the publication of any potentially identifiable images or data included in this article.

## Author contributions

XZ: made substantial contributions to the conception of the work and revised the work critically. YJ: made substantial contributions to the conception, data collection and analysis of the work and drafted the work. All authors contributed to the article and approved the submitted version.

## Funding

This work was supported by the National Natural Science Foundation of China (Grant No. 71772152).

## Conflict of interest

The authors declare that the research was conducted in the absence of any commercial or financial relationships that could be construed as a potential conflict of interest.

## Publisher’s note

All claims expressed in this article are solely those of the authors and do not necessarily represent those of their affiliated organizations, or those of the publisher, the editors and the reviewers. Any product that may be evaluated in this article, or claim that may be made by its manufacturer, is not guaranteed or endorsed by the publisher.
